# Guidance in breast-conserving surgery: tumour localization *versus* identification

**DOI:** 10.1093/bjs/znac409

**Published:** 2022-12-14

**Authors:** Martha S Kedrzycki, Daniel S Elson, Daniel R Leff

**Affiliations:** Department of Surgery and Cancer, Imperial College London, London, UK; Department of Breast Surgery, Charing Cross Hospital, Imperial Healthcare Trust, London, UK; Department of Surgery and Cancer, Imperial College London, London, UK; Hamlyn Centre, Imperial College London, Institute of Global Health Innovation, London, UK; Department of Surgery and Cancer, Imperial College London, London, UK; Department of Breast Surgery, Charing Cross Hospital, Imperial Healthcare Trust, London, UK

## Introduction

In breast-conserving surgery (BCS), the tumour is removed with the goal of preserving as much healthy breast tissue as possible. Breast conservation comes with a risk of positive resection margins, an independent predictor of ipsilateral tumour recurrence, necessitating reoperation^1^. Contemporary data from the UK Get it Right First Time^1^ suggest high average reoperation rates of around 19 %. Current tumour localization techniques can only guide surgeons to the tumour epicentre, but fail to provide identification of the boundary between tumour and normal tissue. Imaging techniques, such as intraoperative ultrasonography (IOUS), intraoperative MRI (iMRI) or fluorescence-guided surgery (FGS), enable visualization of the tumour in its entirety and may provide improved operative precision^2–5^.

## Methods

In April 2022, a literature review was performed exploring localization and identification modalities in BCS. The PubMed electronic database was searched for the highest-quality evidence available for each modality.

## Results

### Tumour localization

Localization techniques involve radiological insertion of a tumour guidance system, followed by surgical resection. These identify/mark the tumour core, which offers surgeons an approximate geolocation, yet they are unable to provide information about the tumour extent or tumour–normal tissue interface, and are therefore associated with a high average reoperation rate^1^. By definition, all localization techniques depend on accurate identification of the target lesion by the radiologist. Challenges include localization and mapping of ductal carcinoma *in situ* that is not detected by ultrasound imaging^6^. Similarly, lobular carcinoma is difficult to detect on mammography and ultrasound examination, and thus MRI guidance is often required^6^.

In the standard wire-guided localization (WGL) technique, a wire is placed in the core of the tumour. Bracketing wires can be used to describe the approximate size of the lesion or multiple lesions within proximity^2,7,8^. Patients, however, find wires uncomfortable, they are prone to displacement and need to be inserted immediately before surgery. This strain on radiology services disrupts patient flow between imaging departments and theatres, delaying operations^2,7,8^.

A variety of alternative localization techniques have been developed to address these flaws. Radio-occult lesion localization (ROLL) comprises injection of a radioactive colloid into the tumour, whereas a radioactive seed is implanted in radioactive seed localization (RSL)^2,3,7,8^. A Cochrane Review^7^ confirmed that WGL is comparable to RSL and ROLL in terms of positive margins and reoperation rates, but the latter are associated with improved patient comfort, hospital flow, or surgical precision. More recently, a ferromagnetic seed (Magseed^®^; Endomag, Cambridge, UK), a reflector to electromagnetic radar signals (SAVI SCOUT^®^; Meritmedical, South Jordan, UT, USA), a radiofrequency tag (Localizer™; Hologic, Marlborough, MA, USA), and an electromagnetic signature for triangulation (Elucent Smart Clip™; Elucent Medical, Eden Prairie, MN, USA; NCT04604561) have entered the market^2,3,8–10^. Seldom used techniques include cryo-assisted localization (freezing) and carbon track localization (intralesional carbon injection), which enable tactile and visual feedback, respectively^8^. Seeds are somewhat difficult for localization of multifocal lesions in close proximity (less than 2 cm), as independent signals would be indistinguishable^2^. Thus, localization techniques help pinpoint the tumour core; but they fail to provide a visual representation of the healthy cancer tissue boundary (*Table S1*).

### Tumour identification

There is an urgent need for techniques that can identify, on a macroscopic scale, tumour location, size, and invasiveness, thus demarcating where disease stops and healthy breast starts. Potential imaging solutions include IOUS, iMRI or FGS^3–5^. IOUS is highly operator-dependent and iMRI (preoperative MRI and intraoperative optical imaging) requires the patient to be in the same position as during the preoperative scan. Both delay the operation, while scanning takes place, so FGS might be a more suitable option^2^ (*Table S1*).

During FGS, a near-infrared dye is administered that targets the tumour, and a light source and dual colour and infrared camera system are used to detect the signal, with the images being displayed on a screen. The images are provided in real time and are not dependent on the skill of the operator nor patient position. As infrared light is used, the healthy tissue–tumour border can be visualized without affecting the surgeon’s view of the operative site. Anecdotal evidence suggests a penetration depth of 4 mm in FGS, which enables both intraoperative guidance and provision of an adequate margin^11,12^. Furthermore, a lack of fluorescence in the resection cavity could be used to verify the adequacy of resection.

There have been multiple studies using US Food and Drug Administration (FDA)-approved non-selective fluorophores, such as indocyanine green (ICG). After injection, ICG leaks into tumours as a result of the enhanced permeability and retention (EPR) effect that capitalizes on the tumour’s porous vasculature and compromised lymphatic outflow. This passive mechanism of action, however, lacks accuracy. The most recent study^11^ combining image analysis with texture metrics surpassed results of previous studies, achieving a sensitivity of 66–82% and specificity of 90–93%.

**Fig. 1:**
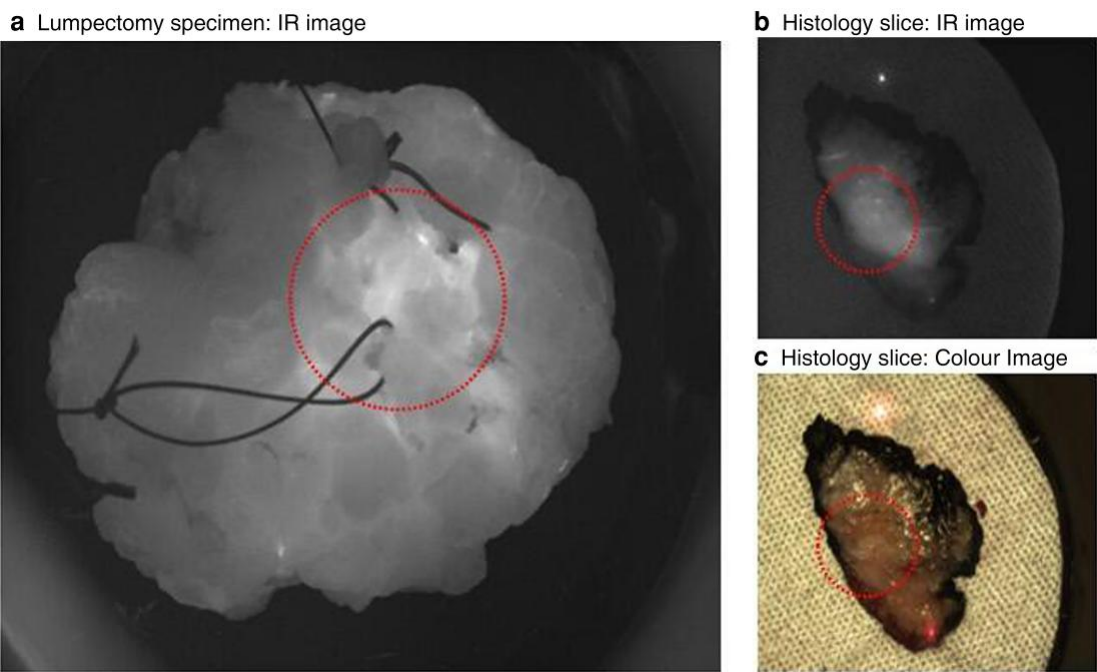
**Images of specimen ex-vivo and during histopathology processing. a** Lumpectomy specimen viewed through an infrared (IR) camera; area encircled is tumour found 1 mm deep to surface. **b** IR and **c** colour images of histology slice, cut from medial to lateral, left-side anterior, right-side posterior; tumour is encircled (Research Ethics Commitee 19/LO/0927)^11^.

The advent of targeting fluorophores has made the delineation between tumour and normal tissue clearer (*Fig. 1*). Eleven targeting fluorophores (8 targeting receptors and 3 targeting enzymes) are currently undergoing clinical trials, and more are being developed^13^. Of these, eight are currently undergoing trials in BCS, with favourable results. For example, bevacuzimab-800 (vascular endothelial growth factor), LUM015 (cathepsin), and EC17 (folate) were able to identify 100% of the lesions to be resected, with sensitivities of 88–98, 100, and 100%, respectively^14–17^. These targeting fluorophores need to gain FDA approval; however, many are already in the latter stages of clinical trials^18,19^.

## Discussion

A plethora of options exist to assist tumour localization for BCS, but none have successfully eliminated the risk of positive margins and reoperation, or enabled tumour identification *in vivo*. Tumour identification techniques such as FGS are in their infancy, but show promise in enhancing operative precision by improving delineation of the boundary between tumour and healthy tissue. FGS has many barriers to overcome before clinical adoption. Because of the heterogeneity of breast cancer in terms of both genotype and phenotype, it is unlikely that overexpression of one protein will be representative of all cancers. Arguably, the ideal scenario would be to use a combination of multiple targeting fluorophores to target various proteins or combination with a margin assessment technique^13^.

Margin assessment techniques cannot guide surgeons to the tumour itself. Rather, they are used to assess the specimen and/or cavity to verify microscopic margins. Immediate cytopathology techniques such as frozen section and imprint cytopathology provide visual demarcation of the tumour–healthy tissue margin. Clinical studies suggest that frozen-section analysis can reduce positive margin rates, but few centres have the resources to facilitate this time-sensitive, resource-intense, and costly technique^20^. Novel margin assessment technologies include optical and bioimpedance techniques. They provide information on tissue composition or light diffraction, but have proven problematic with regard to either limited depth, probe–tissue contact artefacts or spatial misalignment/misregistration^18^. Additionally, the majority of them analyse the cavity after resection, provide complex read-outs, and are slow^18^. Furthermore, as none of these margin assessment techniques were designed to guide resection, but rather to ensure clear margins at a microscopic level, they are of limited use in the initial steps of BCS.

Combining these novel margin assessment methods with FGS, which, unlike other identification modalities, is instantaneous and not dependent on patient position or operator skill level, would very likely enable a satisfactory rim of healthy tissue to be obtained. Not only will surgeons have real-time guidance on a macroscopic scale using FGS, but they will be able to further verify the resection at a microscopic level using these novel margin techniques.

## Supplementary Material

znac409_Supplementary_Data

## Data Availability

Data-sharing is not applicable to this article as no new data were created or analysed in this study.
