# Supplementary material for: Guidance in breast-conserving surgery: tumour localization versus identification
Source: Br J Surg. 2022 Dec 14;110(8):920–2. doi: 10.1093/bjs/znac409 (PMC10361673; doi:10.1093/bjs/znac409)
Supplement: znac409_Supplementary_Data [file znac409_supplementary_data.docx]

**Guidance In Breast Conserving Surgery: Tumour Localisation Versus Identification**

**Authors:**

Martha S. Kedrzycki MSc MRCS^1,2^, Daniel S. Elson MPhys PhD^1,3^, Daniel R. Leff FRCS PhD MS (Hons)^1,2^

**Author Affiliations**

^1^Imperial College London, Department of Surgery & Cancer, London, W2 1NY, UK

^2^Charing Cross Hospital, Department of Breast Surgery, Imperial Healthcare Trust, London, W6 8RF, UK

^3^Imperial College London, Hamlyn Centre, Institute of Global Health Innovation, London, SW7 2AZ, UK

**Corresponding author.**

Martha S. Kedrzycki

Clinical Research Fellow

16 S Wharf Rd, Paddington, London W2 1PF

+447706265099

[martha.kedrzycki@imperial.ac.uk](mailto:martha.kedrzycki@imperial.ac.uk)

**ORCID ID** 0000-0002-3028-5743

**Twitter** @Glow1Doc

**Supplementary Material:**

**Table S1** *pag* 3

**Table S1.** Emerging Tumour Localization and Identification Techniques

| **Technique** | **Mechanism of Action** | **Advantage** | **Disadvantage** | **Positive Margin Rates** |
| --- | --- | --- | --- | --- |
| **Localization** | | | | |
| Wire Guidance Localization  (WGL) ^A,C,D^ | N/A | -inexpensive  -widely accepted  -no need for additional apparatus/ probes  -technique of choice in multifocal lesions within <2cm proximity of one another  -non-ionizing | -disrupts workflow  -uncomfortable for patients  -prone to displacement  -requires insertion <24h pre-op  -trajectory of wire (often coming out at superior or lateral breast) may not be optimal for incision planning  -cannot aid in SLNB | 12.0-23.0% (3,5,7,8) |
| Radio-occult Lesion Localization  (ROLL) ^A,B^ | Radioactive Signal | -workflow efficiency  -can combine with sentinel node localization (SNOLL) | -radioactive (sourcing, hospital infrastructure, USNRC)^6^  -needs to be done <24h pre-op  -requires probe | 17.2% (7,8) |
| Radioactive Seed Localization  (RSL) ^A-D^ | Radioactive Signal | -long half-life of I^125^ (4 months) thus can be used over NACT  -can be used for SLNB  -can be used on its own or in combination with Tc^99^ for SLNB | -radioactive (sourcing, hospital infrastructure, USNRC)^6^  -migration if placed in hematoma  -maximum between implantation and surgery is 1 week  -if multifocal, need >2cm distance between seeds to achieve independent signals  -requires probe  -MRI incompatible | 11.0-11.7%. (3,7,8) |
| Magseed ^B^ | Magnetic Signal | -can be implanted indefinitely prior to surgery thus can be used over NACT  -can be used on its own or combined with Magtrace for SLNB  -non-ionizing | -magnetic interference from surgical instruments  -if multifocal, need >2cm distance between seeds to achieve independent signals  -4cm depth of detection  MRI incompatible | 6.3-9.0% (8) |
| SAVI SCOUT^D^ | Radar Signal | -can be implanted indefinitely prior to surgery (although usually <30 days) thus can be used over NACT  -compatible with MRI  -can be used for SLNB  -non-ionizing | -expensive  -depth limit of 6cm  -if multifocal, need >2cm distance between seeds to achieve independent signals  -requires probe  -MRI incompatible | 7.0% (3) |
| Hologic Localizer | Radiofrequency Signal | -signal up to 6cm  -can additionally target axillary lymph nodes  -can use bracketed seeds to provide size of lesion  -can be placed indefinitely beforehand | -if multifocal, need >2cm distance between seeds to achieve independent signals  -difficulty advancing introducer needle through dense glandular tissue into lesion  -if deployed incorrectly, need to resect both seeds  -MRI incompatible | 8.7- 9.6% (8,9) |
| Elucent Smart Clip | Electromagnetic Signal | -triangulates exact location of seed (distance, depth, direction)  -can be implanted weeks before  -up to 3 clips can be placed  -can be used for SLNB | -affected by electromagnetic instruments (i.e. metallic retractors)  -requires probe  -MRI incompatible | Undergoing Clinical Trial  NCT04604561 (10) |
| Cryo-Assisted Localization  (CAL) ^C^ | Tactile Feedback | -inexpensive  -non-ionizing | -can only provide ‘ice ball’ around probe  -requires immediate operation after intervention  -disrupts workflow  -cannot aid in SLNB | 28.2% (8) |
| Carbon Track Marking | Visual Feedback | -inexpensive  -can be injected up to 1 month before surgery  -non-ionizing | -can only provide visual guide at injection site  -cannot use in lesions which are close to the skin, large, or multifocal  -resistance on slicing  -granuloma formation (mimics BC on scan)  -cannot aid in SLNB | Unknown  (only anecdotal evidence available) |
| **Identification** | | | | |
| Intraoperative Ultrasound  (IOUS) ^C^ | Echoic images | -inexpensive  -provides information on size, invasiveness, and margin status in solid tumors  -can improve visibility by using pre-operative hydrogel marker or hematoma  -can be used to assess  -saves patients discomfort of localising techniques | -operator dependent  -time consuming  -increased intraoperative excision rate  -cannot visualize DCIS  -technical difficulties if irregular shape or air below probe  -false positives if multiple biopsy sites  -cannot aid in SLNB | 5.4% (8) |
| Cherenkov Radiation | Electromagnetic Radiation Signal | -radioisotopes ^18^F or Na^131^I can be used  -can be used to interpret tissue blood volume, oxygen saturation, and major vessels\-saves patients discomfort of localising techniques | -expensive  - poor sensitivity (due to scattering and absorption)  -low penetration in tissue  -radioactive (sourcing, hospital infrastructure, USNRC)^6^  ^-^weak luminescence intensity  -light flashes in patients  -cannot aid in SLNB | unknown |
| Optical Scanning  (OS) ^B^ | Preoperative Supine MRI Image and Intraoperative Optical Image | -enables 3D image reconstruction of tumor  -information on size and location  -noncontact (does not require probe)  -saves patients discomfort of localising techniques | -not yet clinically approved (NCT019229395)  -takes long time to scan entire surface  -any MRI contraindications  -necessitates additional pre-operative imaging (MRI)  -false readings if patient MRI position does not match position intraoperatively  -cannot aid in SLNB | 11.8-12.0% (5,8) |
| Fluorescence Guided Surgery  (FGS) ^B^ | Fluorescent Signal | -noncontact (does not require probe)  -Independent of operator  -Real-time visual feedback of tumor location, size, invasiveness  -can be combined with infrared sentinel node mapping  -provide chemical tagging of tumor  -can visualize multiple lesions  -saves patients discomfort of localising techniques | -not yet clinically approved  -success dependent on amount of target receptors/ enzymes being present  -potential side effects of dyes being used  -cannot aid in SLNB | *Studies less than 50 patients.  PMR not available.  Identification rate 100% with  becacizumab800 and LUM015 |

Abbreviations: BC, breast cancer, I^125^, Iridium 125, TC^99^, Technitium 99, USNRC, United States Nuclear Regulatory Committee, SNOLL, sentinel node and occult lesion localization, SLNB, sentinel lymph node biopsy, H&E, Hematoxylin and Eosin; IHC, Immunohistochemistry, Sn= sensitivity, Sp= Specificity

A: as per Cochrane review, there is no statistically significant difference between WGL, ROLL, and RSL

B: as per Davey et al., no statistically significant difference between ROLL, RSL, ML, CAL, OS, or FGS (when non-targeting dye used)

C: as per Davey et al., statistically significantly better than WGL

D: as per Mallory et al., no significant difference between WGL, RSL, SaviScout
